# ^18^F-FDG PET-Based Imaging of Myocardial Inflammation Following Acute Myocardial Infarction in a Mouse Model

**DOI:** 10.3390/ijms21093340

**Published:** 2020-05-08

**Authors:** Praveen Vasudevan, Ralf Gäbel, Jan Stenzel, Joanna Förster, Jens Kurth, Brigitte Vollmar, Bernd Joachim Krause, Hüseyin Ince, Robert David, Cajetan Immanuel Lang

**Affiliations:** 1 Department of Cardiac Surgery, Rostock University Medical Center, 18057 Rostock, Germany; Praveen.Vasudevan@med.uni-rostock.de (P.V.); Ralf.Gaebel@med.uni-rostock.de (R.G.); 2 Department of Life, Light and Matter, Rostock, University of Rostock, 18057 Rostock, Germany; 3 Core Facility Multimodal Small Animal Imaging, University Medical Center, 18057 Rostock, Germany; jan2.stenzel@gmail.com (J.S.); Joanna.Foerster@med.uni-rostock.de (J.F.); 4 Department of Nuclear Medicine, Rostock University Medical Center, 18057 Rostock, Germany; Jens.Kurth@med.uni-rostock.de (J.K.); Bernd.Krause@med.uni-rostock.de (B.J.K.); 5 Rudolf-Zenker-Institute for Experimental Surgery, Rostock University Medical Center, 18057 Rostock, Germany; Brigitte.Vollmar@med.uni-rostock.de; 6 Department of Cardiology, Rostock University Medical Center, 18057 Rostock, Germany; Hueseyin.Ince@med.uni-rostock.de (H.I.); Cajetan.Lang@med.uni-rostock.de (C.I.L.)

**Keywords:** FDG-PET, imaging inflammation, acute myocardial infarction, mouse model, image analysis

## Abstract

Cellular inflammation is an integral part of the healing process following acute myocardial infarction and has been under intense investigation for both therapeutic and prognostic approaches. Monocytes and macrophages are metabolically highly active and show increased uptake rates of glucose and its analog, ^18^F-FDG. Yet, the specific allocation of the radioactivity to the inflammatory cells via positron emission tomography (PET) imaging requires the suppression of glucose metabolism in viable myocardium. In mice, the most important model organism in basic research, this can be achieved by the application of ketamine/xylazine (KX) for anesthesia instead of isoflurane. Yet, while the consensus exists that glucose metabolism is effectively suppressed, a strategy for reproducible image analysis is grossly lacking and causes uncertainty concerning data interpretation. We introduce a simple strategy for systematic image analysis, which is a prerequisite to evaluate therapies targeting myocardial inflammation. Mice underwent permanent occlusion of the left anterior descending artery (LAD), inducing an acute myocardial infarction (MI). Five days after MI induction, 10MBq ^18^F-FDG was injected intravenously and a static PET/CT scan under ketamine/xylazine anesthesia was performed. For image reconstruction, we used an algorithm based on three-dimensional ordered subsets expectation maximization (3D-OSEM) followed by three-dimensional ordinary Poisson maximum a priori (MAP) reconstruction. Using this approach, high focal tracer uptake was typically located in the border zone of the infarct by visual inspection. To precisely demarcate the border zone for reproducible volume of interest (VOI) positioning, our protocol relies on positioning VOIs around the whole left ventricle, the inferobasal wall and the anterolateral wall guided by anatomical landmarks. This strategy enables comparable data in mouse studies, which is an important prerequisite for using a PET-based assessment of myocardial inflammation as a prognostic tool in therapeutic applications.

## 1. Introduction

According to the World Health Organization, coronary artery disease (CAD) is the world’s biggest killer, accounting for almost 10 million deaths per year worldwide. CAD is defined by atherosclerosis of the coronary arteries leading to a reduction of the blood flow to the myocardium [1]. Plaques involved in this atherosclerotic process may rupture and form a blood clot, which in turn occludes the respective coronary artery and causes an acute myocardial infarction (AMI). Modern interventional and surgical approaches have drastically reduced the mortality of AMI. Yet, the irreversible loss of working cardiomyocytes eventually results in congestive heart failure over time in many patients [2].

Therefore, strategies to minimize the loss of viable myocardium and to support the healing process following ischemic damage of the heart have been under intense investigation during the last decades. Early trials performed since the 1970s have aimed to reduce myocardial damage following AMI by administrating unspecific inhibitors of inflammation such as corticosteroids, ibuprofen and indomethacin [3]. None of these concepts aiming at the unspecific reduction of myocardial inflammation have proven successful in clinical trials [4]. Despite negative results in early studies, these studies paved the way for more specific approaches targeting at distinct leucocyte subpopulations.

Recent evidence reveals that it is rather the amplitude and duration of inflammation and the timely and spatial resolution within the heart, which defines the quality of the scar following MI and the amount of tissue loss [5] than the mere extent of inflammation. Inflammation is an integral part of the complex process of myocardial healing and predominantly orchestrated by distinct monocyte subpopulations. A simplified concept distinguishes two functional subpopulations based on their immunophenotype, namely the extent of “Ly-6C” expression. Ly-6C^high^ monocytes dominate the inflammatory phase, whereas Ly-6C^low^ plays a central role in the proliferative phase by stimulating fibroblasts, angiogenesis and collagen formation [6].

For this reason, the concept of the unspecific suppression of inflammation has changed to a targeted modulation of cellular inflammation for both therapeutic and prognostic approaches [5]. Therapeutic modulations of these monocyte subpopulations can be assessed in post-mortem experiments by flow cytometry in animal models. In contrast, clinical translation of these concepts requires non-invasive experimental setups.

^18^F-FDG PET is well established for imaging inflammation in patients and animals due to high levels of glucose transporters and thus increased ^18^F-FDG uptake of activated inflammatory cells [7]. Clear allocation of focal ^18^F-FDG accumulation requires low rates of baseline glucose in the tissue of interest, which hampers straightforward application of this method to the metabolically highly active heart. This issue has been solved by the introduction of specific protocols for suppressing glucose uptake in cardiomyocytes in both patients and mice [5,8,9,10]. The latter play an important role in this field of research, as the mouse is a key small animal model in basic research and enables the correlation of molecular biology and in-vivo imaging approaches. The use of ketamine/xylazine (KX) for anesthesia has been shown to suppress myocardial ^18^F-FDG uptake effectively [5,9,10]. In our own previous work, a reduction of almost 90% could be achieved in healthy mice [11]. This concept is based on the observation that KX for anesthesia reduces serum insulin levels in rodents and thus prevents translocation of glucose transporter (GLUT) 4 to membranes of cardiomyocytes [12]. In contrast, glucose influx in activated leucocytes depends rather on GLUT 1 and GLUT3. Both transporters are expressed and translocated independently of insulin [13]. This concept has been substantiated by Lee et al. who could attribute ^18^F-FDG uptake to inflammatory CD11b^+^ myeloid cells in a mouse model of myocardial infarction [5]. Yet, the use of this approach for the assessment and quantification of cellular inflammation in the context of therapy monitoring is currently hampered by the lack of strategies for image analysis. In this protocol we describe an approach for measuring of the focal tracer uptake based on positioning volumes of interest (VOI) around the whole left ventricle, the inferobasal and the anterolateral wall. This strategy will help researchers to generate comparable data when using ^18^F-FDG PET for imaging cellular inflammation and its modulation post-acute myocardial infarction.

## 2. Results

**Figure 1:**
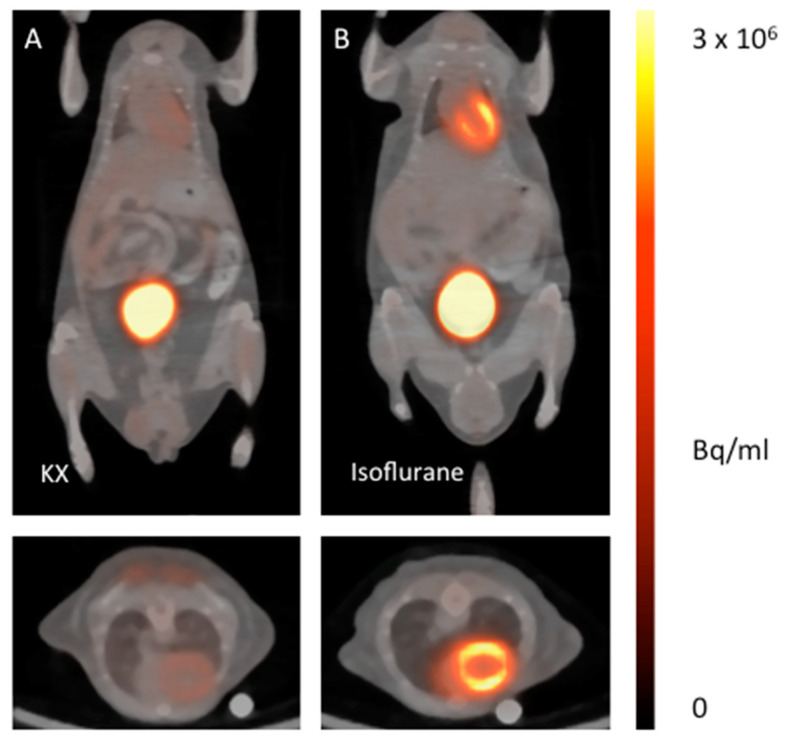
^18^F-FDG PET/CT fusion images of healthy mice anesthetized with ketamine/xylazine (**A**) in comparison with isoflurane (**B**). The upper images show representative coronal planes, the lower images the corresponding axial plane.

**Figure 2:**
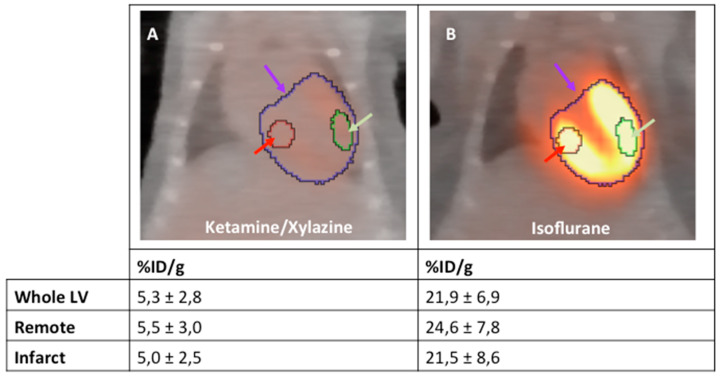
^18^F-FDG PET/CT fusion images of healthy mice anesthetized with ketamine/xylazine (**A**) and isoflurane (**B**); *n* = 2 per group. Representative standard VOIs are placed in whole LV (purple arrow), remote (red arrow) and infarct region (green arrow).

The ketamine/xylazine protocol for anesthetizing healthy mice suppresses glucose metabolism of viable cardiomyocytes in healthy mice significantly compared to isoflurane (Figure 1 and Figure 2). When setting the ranges of the color bars to identical minimum and maximum values, the effect can be clearly visualized.

**Figure 3:**
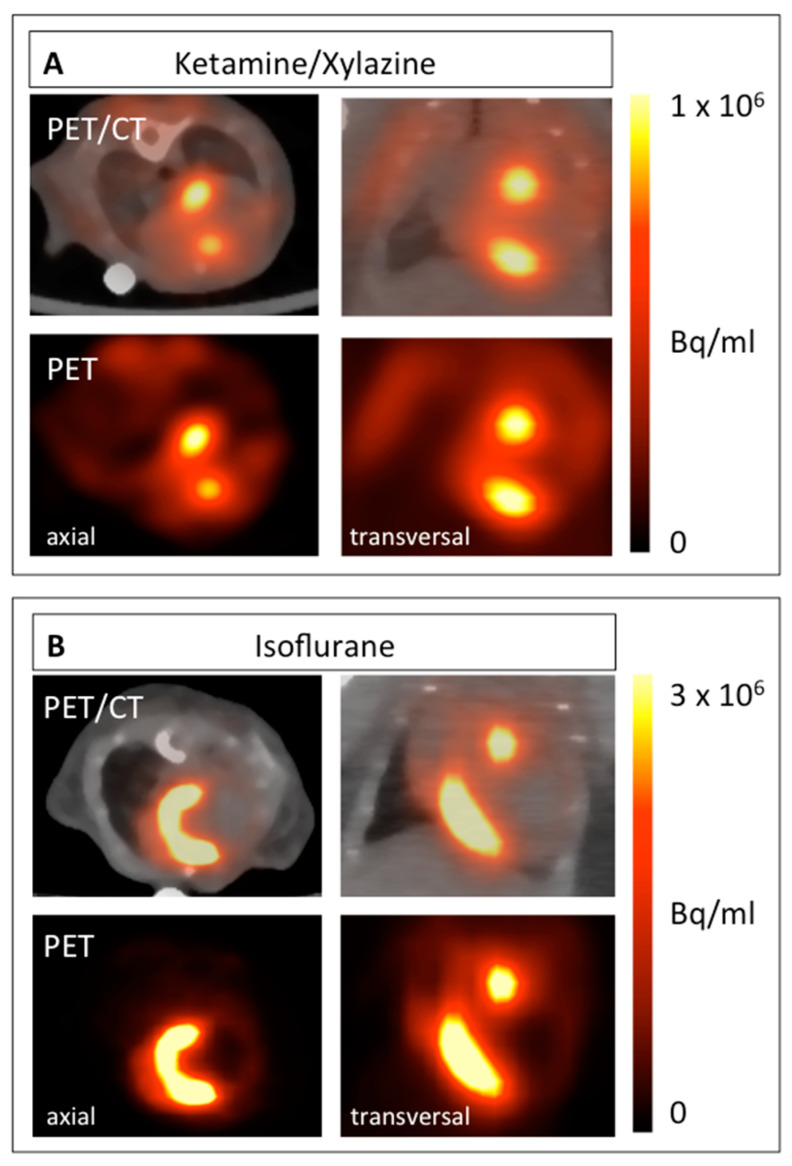
^18^F-FDG PET images of mice 5 days after MI induction anesthetized with ketamine/xylazine (**A**) in comparison with isoflurane (**B**). Both axial (left) and coronal planes (right) are shown. The respective PET image is shown under each PET/CT fusion image.

This protocol can be used to visualize and quantify infiltrating monocytes in the process of healing following acute myocardial infarction. When glucose metabolism is suppressed, the highest focal tracer accumulation can be detected within the border zone of the infarct (Figure 3A). In contrast, when mice are anesthetized with isoflurane, ^18^F-FDG accumulates predominantly within the viable myocardium (Figure 3B).

**Figure 4:**
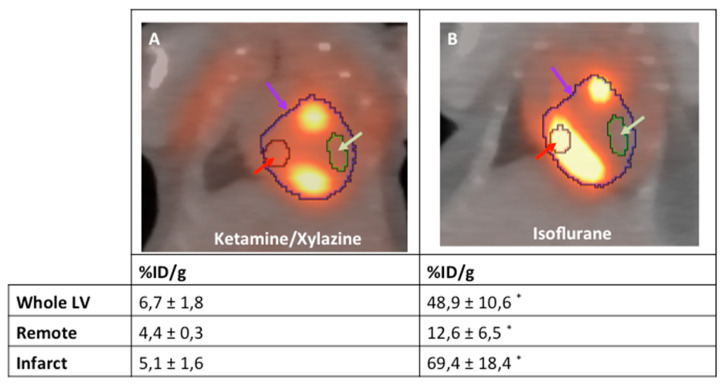
Representative examples of the analysis strategy underlying the protocol for both mice anesthetized with isoflurane (**A**) and ketamine/xylazine (**B**) 5 days after MI induction (*n* = 4 per group). The “entire left ventricle” VOI reflects the global FDG uptake of the LV (purple arrow). The “remote” VOI was positioned in the inferobasal wall and reflects viable myocardium (red arrow). The “infarct” VOI reflects infarct tissue and contains almost no cardiomyocytes (green arrow). *: *p* < 0.05 compared to animals anesthetized with ketamine/xylazine. Values are presented as mean ± SD. Values are presented as mean ± SD. *p*-values were calculated using the student t-test.

**Figure 5:**
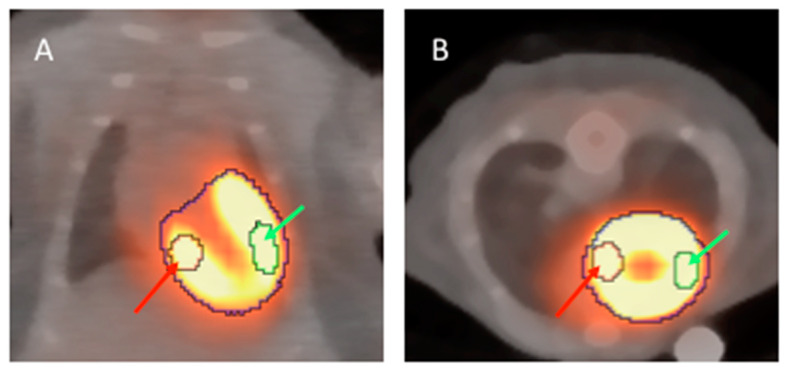
^18^F-FDG PET/CT fusion images of a healthy mouse anesthetized with isoflurane. The left ventricle can be clearly identified in both coronal (**A**) and axial (**B**) planes. VOIs of 5 µL are placed in the inferobasal wall (red arrow) and the anterolateral wall (green arrow).

As the exact extent of the border zone cannot be determined the pattern of ^18^F-FDG accumulation can only be described qualitatively from the mere PET/CT images (Figure 3). Therefore, we developed a protocol to quantify this change in the ^18^F-FDG upake pattern relying on an indirect approach. To this end, VOIs were positioned around the entire left ventricle (LV), the inferobasal wall and the anterolateral wall. These regions can be localized relatively easily from the PET/CT images as shown in Figure 4. As defining these VOIs in infarcted animals is difficult, a healthy animal anesthetized with isoflurane was used for VOI definition. By importing these VOIs from healthy animals for image analysis, the respective regions of the LV in infarcted animals could be easily reproduced (Figure 5).

## 3. Discussion

Cellular inflammation is an integral part of the healing process after myocardial infarction and thus an attractive target for both therapeutic and diagnostic approaches. Non-invasive imaging tools are indispensible for translation of such approaches into clinical practice. Therefore, a protocol for the imaging of cellular inflammation in mice based on ^18^F-FDG PET has been evaluated by us and others [5,9,10,11]. The mouse is of specific interest due to its relevance in basic research and the availability of imaging modalities, which can be used for both mice and men.

The prerequisite for specific imaging of inflammation is an effective protocol for suppression of myocardial glucose uptake, which could be achieved by the use of ketamine/xylazine (KX) for anesthesia in different mouse strains by several groups [5,9,10,11]. In contrast to the consensus on suppressing myocardial glucose uptake, strategies concerning the analysis of obtained images and data interpretation are less coherent. Furthermore, the time point chosen for image acquisition ranges from 3–7 days post MI between available studies [5,9,10]. Therefore, it is important to correlate the imaging data with the respective monocyte or macrophage population, which is dominating the healing process at the respective time point. Both ours and the data obtained by Nahrendorf´s group detected the highest focal ^18^F-FDG accumulations in the border zone five days after MI [5,11]. This can be clearly seen in PET/CT images and correlates well with histological findings [5]. Yet, there is no reproducible VOI positioning strategy available for quantification of radioactivity in the border zone.

Hence, we introduced an indirect approach to describe the ^18^F-FDG distribution pattern by positioning three VOIs into defined areas of the heart. The fist VOI encompasses the whole LV and reflects global glucose metabolism. Then, two representative VOIs of approximately 5 µL are placed into the infarct region (anterolateral wall) and the remote area (inferobasal wall). Both regions can be easily located by from anatomical landmarks in the PET/CT images from a healthy animal anasthetized with isoflurane. We believe that our approach provides a method that helps to obtain a comparable result in the imaging myocardial inflammation by ^18^F-FDG PET/CT. Yet, further research on the glucose metabolism of the different monocyte subpopulations will be important in order to further improve the quality of molecular imaging in this field.

## 4. Limitations

Our strategy for image analysis and interpretation provides an indirect approach for the semi-quantitative assessment of myocardial inflammation. The border zone cannot be quantified by this approach, as it consists of an overlap of both remote and infarcted myocardia. Therefore, our strategy should be complemented by other imaging approaches for studying the border zone. The left and right ventricles as well as the atria cannot be distinguished reliably from CT images. Furthermore, the use of ketamine/xylazine effectively suppresses myocardial glucose uptake making the delineation of the left ventricle impossible in the respective scan. Yet, left ventricular volume can be perfectly assessed from ^18^F-FDG PET scans under anesthesia with isoflurane, as described by Todica et al. [14]. Therefore, an additional scan to obtain an “LV VOI template” using isoflurane is necessary in our approach.

## 5. Materials and Methods

### 5.1. Animal Model

The experiments in this study were approved by the federal animal care committee of the *Landesamt für Landwirtschaft, Lebensmittelsicherheit und Fischerei Mecklenburg-Vorpommern* (local animal protection authority, Germany) (registration no. LALLF M-V/TSD/7221.3-1.1-054/15; approved by 16 February 2018). Mice of the strain 129S6/SvEvTac were bred in the animal facility of the Rostock University Medical Center. Animals used were 12–14 weeks old, had a body weight of about 20 g and had the same access to food and water. Acute myocardial infarction was induced by permanent occlusion of the LAD as described previously [11]. For establishing of the protocol described, at least one healthy animal and two animals with myocardial infarctions should be included. PET imaging was performed 5 days after MI induction.

### 5.2. PET Imaging

In order to obtain images showing the glucose metabolism of the myocardium of a healthy animal, animals with myocardial infarction were anesthetized by inhalation of isoflurane (4% for induction and 1–2.5% maintenance during preparation and scanning). The healthy control is used to define and align the VOIs. For imaging cellular inflammation, the respective mouse was anesthetized by i.p injection of ketamine/xylazine (ketamine 84 mg/kg and xylazine 11.2 mg/kg) 20 min before tracer application. The KX control is used to verify the suppression of glucose metabolism.

Images were acquired on a small animal PET/CT scanner (Inveon MM-PET/CT, Siemens Medical Solutions, Knoxville, TN, USA) according to a standard protocol: 10MBq ^18^F-FDG was injected intravenously via a custom-made micro catheter placed in a tail vein. Mice were imaged after an uptake period of 60 min in a prone position for 20 min. Respiration of the mice was controlled and core body temperature was constantly kept at 38 °C via a heating pad during the imaging procedure. A Feldkamp algorithm was used to reconstruct CT images.

The 3-dimensional (3-D) iterative ordered-subset expectation maximization reconstruction algorithm (3D-OSEM/OP-MAP) with the following parameters was used for reconstruction of the PET images: 4 iterations (OSEM), 32 iterations (MAP), 1.7 mm target resolution and 128 × 128 matrix size. Reconstruction included corrections for random coincidences, dead time, attenuation, scatter, and decay.

### 5.3. PET Image Analysis

Image analysis was performed using an Inveon Research Workplace 4.2 (Siemens, Knoxville, TN, USA). PET and CT images were fused by the use of an automated volumetric fusion algorithm and then verified by an experienced reader for perfect alignment and adjusted when necessary. For qualitative analysis and a comparison of different groups, the activity concentration in the PET images were displayed as “Voxel Intensity” (Bq/mL). For a visual comparison of animals anesthetized with isoflurane and ketamine/xylazine, the scale bar was set from 0 to 3 × 10^6^ Bq/mL (isoflurane) and 1 × 10^6^ Bq/mL (KX) respectively.

The healthy animal anesthetized with isoflurane was used for defining the VOIs, which were used in the following analyses of infarcted animals. The whole left ventricle (LV) shows intense 18F-FDG uptake enabling easy VOI positioning (Figure 5). The VOIs were placed into the respective regions as described below, and then saved into a selected folder:

Global uptake in LV (“whole LV”): A spherical VOI was drawn around the LV and its size adjusted with the “edit” function, using the CT image for correct anatomical positioning. The VOI will typically have a volume of 200 µL.

Infarcted Myocardium: A spherical VOI of 5 µL (+/− 0.1µL) was positioned in the anterolateral wall, based on anatomical landmarks from CT and PET images.

Remote Myocardium: A spherical VOI of 5 µL (+/− 0.1µL) was positioned in the inferobasal wall, based on anatomical landmarks from CT and PET images.

For a semi-quantitative comparison of myocardial ^18^F-FDG uptake of infarcted hearts, the VOIs which have been defined in the healthy animal before were imported. VOIs were adjusted and fine-tuned based on anatomical landmarks from the PET and CT images.

### 5.4. Statistics

The data are presented as mean values ± standard deviation (SD). Student’s *t*-test was used to assess the statistical difference between the groups. Values of *p* < 0.05 were considered statistically significant.

## Abbreviations

FDG	fluorodeoxyglucose
PET	positron emission tomography
KX	ketamine/xylazine
CT	computer tomography
VOI	volume of interest
AMI	acute myocardial infarction
MI	myocardial infarction
%ID/g	% of injected dose per gram
SD	standard deviation
LV	left ventricle
GLUT	glucose transporter
